# PINNet: a deep neural network with pathway prior knowledge for Alzheimer's disease

**DOI:** 10.3389/fnagi.2023.1126156

**Published:** 2023-07-14

**Authors:** Yeojin Kim, Hyunju Lee

**Affiliations:** ^1^Artificial Intelligence Graduate School, Gwangju Institute of Science and Technology, Gwangju, Republic of Korea; ^2^School of Electrical Engineering and Computer Science, Gwangju Institute of Science and Technology, Gwangju, Republic of Korea

**Keywords:** Alzheimer's disease, machine learning, transcriptomics, biomarkers, bioinformatics, protein-protein interaction network, interpretable machine learning

## Abstract

**Introduction:**

Identification of Alzheimer's Disease (AD)-related transcriptomic signatures from blood is important for early diagnosis of the disease. Deep learning techniques are potent classifiers for AD diagnosis, but most have been unable to identify biomarkers because of their lack of interpretability.

**Methods:**

To address these challenges, we propose a pathway information-based neural network (PINNet) to predict AD patients and analyze blood and brain transcriptomic signatures using an interpretable deep learning model. PINNet is a deep neural network (DNN) model with pathway prior knowledge from either the Gene Ontology or Kyoto Encyclopedia of Genes and Genomes databases. Then, a backpropagation-based model interpretation method was applied to reveal essential pathways and genes for predicting AD.

**Results:**

The performance of PINNet was compared with a DNN model without a pathway. Performances of PINNet outperformed or were similar to those of DNN without a pathway using blood and brain gene expressions, respectively. Moreover, PINNet considers more AD-related genes as essential features than DNN without a pathway in the learning process. Pathway analysis of protein-protein interaction modules of highly contributed genes showed that AD-related genes in blood were enriched with cell migration, PI3K-Akt, MAPK signaling, and apoptosis in blood. The pathways enriched in the brain module included cell migration, PI3K-Akt, MAPK signaling, apoptosis, protein ubiquitination, and *t*-cell activation.

**Discussion:**

By integrating prior knowledge about pathways, PINNet can reveal essential pathways related to AD. The source codes are available at https://github.com/DMCB-GIST/PINNet.

## 1. Introduction

Alzheimer's disease (AD) is the most prevalent type of dementia and is distinguished by amyloid beta (Aβ) plaques and neurofibrillary tangles in the brain. Aβ and tau are clinical hallmarks of AD, in which abnormal accumulation precedes neurodegeneration and cognitive impairment in both sporadic and familial AD (Bateman et al., 2012). Although much progress has been made in understanding AD pathology, currently available treatments modify only symptoms, such as cognitive and behavioral dysfunction (Yiannopoulou and Papageorgiou, 2020). Thus, in the pre-dementia phase, prior to the appearance of clinical symptoms, the early detection of AD using biomarkers or neuroimaging data is essential for treatments to slow progression of AD (Huynh and Mohan, 2017; Lim et al., 2022). Recently, AD has been increasingly recognized as a systemic disease, supported by numerous studies that have uncovered a peripheral mechanism for AD progression (Zhang et al., 2013; Morris et al., 2014). Brain-derived Aβ can be cleared in the periphery by monocytes, which is boosted by the immune system (Cheng et al., 2020). Multiple peripheral inflammatory markers were elevated in AD patients (Lai et al., 2017), and it was showed that inflammation also plays an essential role in AD development. Furthermore, Urayama et al. (2022) reported a significant reduction of Aβ plaque development by 40–80% and improvement in memory performance through exchanging the whole blood in AD mice with normal blood. However, the exact mechanism of how blood exchanges reduce amyloid pathology remains unclear.

Several studies based on gene expression data have been conducted to find biomarkers for AD. Puthiyedth et al. (2016) analyzed microarray gene expression data of 161 post-mortem brain samples across six brain regions and identified new AD candidate genes and 23 non-coding features. Xu et al. (2018) analyzed gene expressions from brain tissues by constructing a transcriptomic network and demonstrated that activation of 17 hub genes, including YAP1, at the early stage could promote AD. Although gene expression data of brain tissues obtained from post-mortem autopsy revealed important molecular mechanisms regarding AD, their clinical application is limited due to their invasiveness. Instead, studies based on blood gene expression data have been conducted for early diagnosis of Alzheimer's disease. Li et al. (2017) detected leukocyte-specific expression changes in peripheral whole blood gene expression data. They found that differentially expressed genes were associated with Wnt signaling pathways and mitochondrial dysfunction, suggesting a significant overlap in brain area expression profiles. However, few studies have compared the transcriptomic signatures of AD between blood samples and brain samples.

Many deep learning methods have been applied to biomedical domains and showed improved performances for various problems such as predictions of properties of DNA sequences, protein structure prediction, and disease classification (Luo et al., 2019; Mostavi et al., 2020; Senior et al., 2020). In these methods, weights in neural networks are optimized for a given objective function, but they do not necessarily reveal a group of neurons and input features related to a given biomedical problem. Recently, several studies have started searching for a model to obtain outcomes with explainable mechanisms (Kuenzi et al., 2020; Lee et al., 2020). Mao et al. (2019) proposed an algorithm that optimizes gene expression data decomposition by incorporating biological knowledge through the addition of constraints to standard singular value decomposition methods. Furthermore, (Xing et al., 2021) presented a graph-based model that extracts hierarchical gene-module features from co-expression graphs using weighted correlation network analysis. Both approaches aim to improve the interpretability of models by introducing biological knowledge to capture relevant biological processes. Nonetheless, although performance comparisons have been conducted for models incorporating biological prior knowledge, a comprehensive evaluation focusing on interpretability, particularly identifying specific genes crucial to the model's predictions, remains limited in the previous studies. Without clear interpretability, it becomes challenging to translate the findings of the models into meaningful biological or clinical insights.

This study proposes a pathway information-based deep neural network (PINNet) to predict AD using a gene expression dataset from the brain and blood. The motivation for the PINNet is incorporating the explicit gene relationships via pathway information, as well as capturing implicit gene relationships through the fully connected layer in the model's structure. To determine the model's interpretability across various pathway contexts, we considered two sources of pathway information: Gene Ontology (GO), a literature-curated reference database with a hierarchical structure, or the Kyoto Encyclopedia of Genes and Genomes (KEGG), an alternative pathway ontology containing dynamics and interaction between the genes. The GO ontology design is interrelated through a hierarchical parent-child relationship, enabling us to examine functional clusters at different scales. KEGG represents networks of interacting molecules responsible for specific biological functions. In PINNet, pathway information was included in the structure of the model, thereby enhancing interpretation. The model was interpreted using Deep SHAP (Lundberg and Lee, 2017), which allows us to identify important predictor genes in the model. Thus, we evaluated the performance of PINNet models in blood and brain gene expression samples for two pathway databases. When we examined gene signatures of blood and brain that played essential roles in each predictive model, contributing genes in PINNet were highly prominent in known sets of AD-related genes. The main contribution of this study is the effective integration of biological prior knowledge into a deep learning model, thereby improving both predictive performance and interpretability. Notably, by integrating pathway information from either GO or KEGG, we substantially enhance our model's understanding of AD-related genes and pathways.

## 2. Materials and methods

### 2.1. Preprocessing of data

Gene expression datasets from the prefrontal cortex brain and blood were obtained from GSE33000 (Narayanan et al., 2014) and Alzheimer's Disease Neuroimaging Initiative (ADNI) (adni.loni.usc.edu) (Petersen et al., 2010), respectively. Brain and blood gene expression data were generated using the Rosetta/Merck Human 44k 1.1 microarray (Narayanan et al., 2014) and the Affymetrix Human Genome U 219 array (Petersen et al., 2010), respectively. We used 113 AD and 244 control (CN) samples for the blood dataset. To maintain consistency between the blood and brain datasets, mild cognitive impairment (MCI) samples were excluded from the analysis, as the brain dataset does not contain MCI samples. For the brain dataset, we used 310 AD and 157 CN samples after excluding Huntington's disease samples. The blood and brain datasets consist of 49,386 and 39,328 probes, respectively. The mean imputation was performed on the remaining missing data values in each dataset. We removed the 30% of probes with a low interquartile range (IQR) across samples. To compare gene expression across different platforms, we mapped probe IDs to Entrez IDs using the biomaRt package (Durinck et al., 2009). The probe with the maximum IQR value was selected for multiple probes annotated with the same gene. By using a MinMaxScaler, values in each dataset are preprocessed to fit within the range of 0 and 1. As a result, 13,666 and 12,319 genes remained in the brain and blood datasets, respectively. A total of 8,922 genes were common between the brain and blood datasets, and they were used in this study.

Pathway information was compiled from the GO biological process (BP) (Ashburner et al., 2000) and KEGG (Kanehisa and Goto, 2000) databases obtained from the Molecular Signature Database (MSigDB) (Subramanian et al., 2005). A total of 168 KEGG pathways and 4,026 GO BP were selected. They include ten or more genes among the 8,922 genes used in this study. Using each of the two databases, a pathway information matrix *M*∈ℝ^*m*×*n*^ is constructed, which is a sparse matrix representing the relationship between pathways and genes (Equation 1), where *m* is the number of pathways and *n* is the number of genes.


(1) $$M_{ij}=\{\begin{array}{ll} 0 & j_{gene}\notin i_{pathway}\\ 1 & j_{gene}\in i_{pathway} \end{array}$$

### 2.2. PINNet: a deep neural network with pathway prior knowledge for AD classification

**Figure 1:**
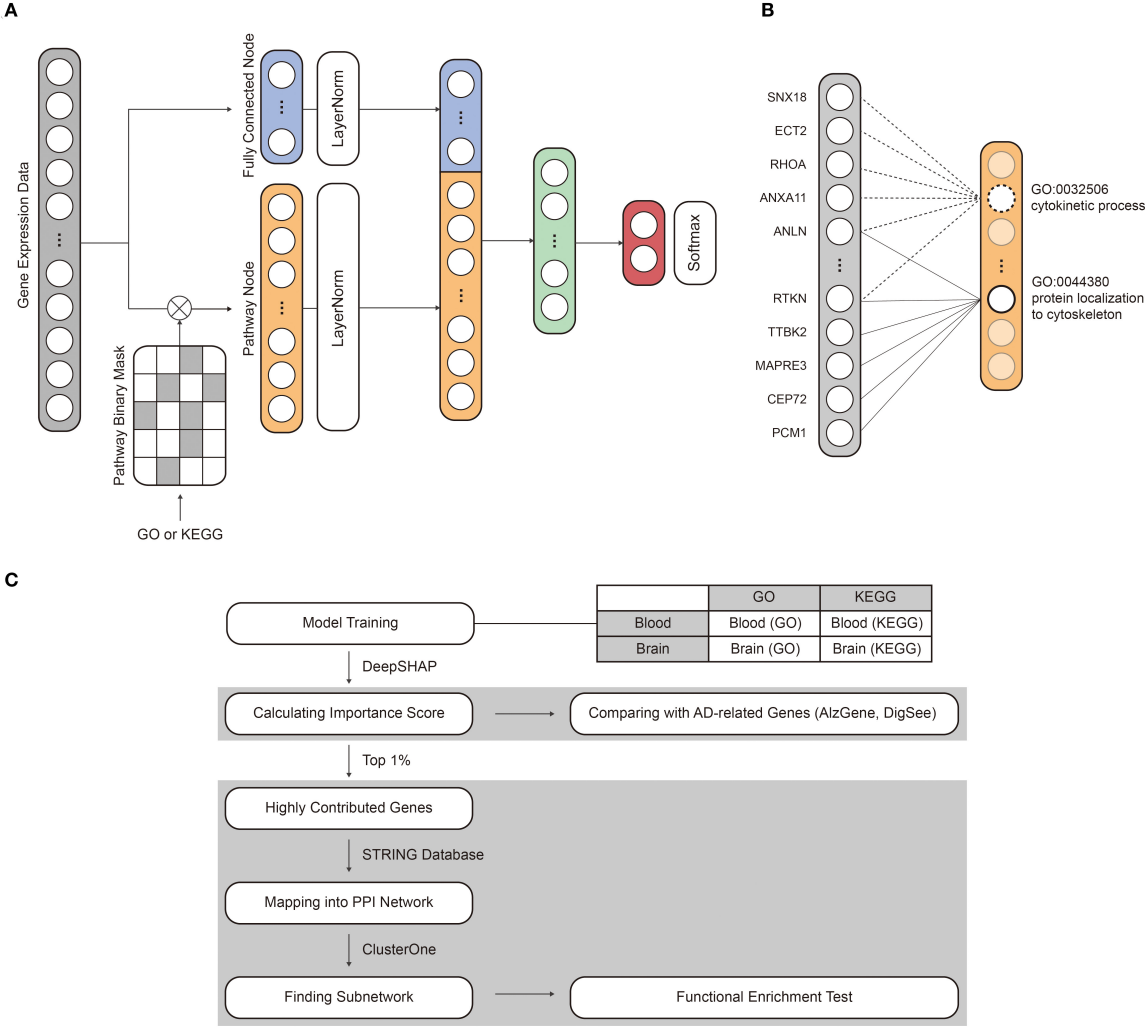
An overview of the model structure and workflow. **(A)** The model consists of an input layer, a pathway layer, a hidden layer, and a softmax layer. The mRNA expression dataset and pathway information were assigned to the input nodes and binary mask matrix, respectively. **(B)** The pathway layer connects the relationship between pathways and genes. **(C)** The interpretability of PINNet was evaluated by comparing importance scores with known AD-related genes. In addition, we identified subnetworks of highly contributed genes and examined related biological processes through a functional enrichment test.

To aid in identifying transcriptional features regulating AD, we developed a deep neural network-based classification model named PINNet, which predicts AD based on pathway information. Figure 1 shows the structure of the PINNet. Contrary to previous deep learning approaches, PINNet does not rely on operating as a black box but instead focuses on prior biological knowledge from the GO or KEGG databases. PINNet consists of four layers: an input layer, a pathway layer, a fully connected hidden layer, and a softmax output layer, and the model is built to predict AD (see Figure 1 and Methods). Blood or brain transcription datasets are fed into the input layer of the model, and the output layer indicates a sample status (AD or CN). We formulated the structure of the model from genes to biological functional groups, enabling biological interpretation. The use of multiple levels of ontology (ranging from 10 to 1,156 genes per pathway) acknowledges the diverse complexity of biological processes. During training, the expression data of each gene are induced to patterns of pathway-level of activities, enabling *in silico* investigation of the biological processes underlying transcriptome-disease association. Genes are partially propagated through the same biological process that contains them, giving rise to functional changes at the pathway level, consequently predicting sample status. Using this design, the PINNet embedded in GO includes 4,026 biological process terms, and the corresponding model for KEGG includes 168 pathways. To incorporate the effect of the pathways, we partially masked the weights of the pathway layer using a binary matrix that represents relationships between pathways and genes. As these neurons' connection is designed to represent biological processes, pathway nodes only receive inputs from the genes included in the pathway. On average, one GO BP is connected to 84 input gene nodes and 42 input gene nodes for KEGG. We normalized pathway nodes with the size of pathways to ensure that the number of genes included in the pathway does not affect the importance of the pathway node. In addition, since the pathway node is very sparsely connected, there is a difference in the range of values of the pathway node and the fully connected node. Therefore, layer normalization was applied to the mini-batch when pathway nodes and fully connected nodes were concatenated. Afterward, they pass through one hidden layer and softmax to predict AD.

### 2.3. Structure and training of PINNet

PINNet is a four-layer deep neural network with biological pathway information as prior knowledge for AD classification. The PINNet comprises four layers: an input layer, a pathway layer, a hidden layer, and an output layer, as shown in Figure 1. Gene expression values of a sample *g*∈ℝ^*n*^ are allocated to input nodes. Thus, the number of input nodes (*n*) is the same as the number of genes (*n* = 8, 922) in the dataset. The pathway layer consists of fully connected nodes *f* and pathway nodes *p*. Pathway nodes *p* represent relations between genes and pathways, and fully connected nodes *f* represent features for all genes regardless that they are included in the pathway. The number of nodes in *p*∈ℝ^*m*^ was the same as the number of pathways used. For instance, *m* is 7,470 when using the GO database and 186 when using the KEGG database. In order to represent the relationship between the gene and the pathway, we obtain pathway nodes *p* as


(2) $$\begin{array}{l} p=tanh\left(\left(\left(W_{p}•M\right)\times g\right)•u\right), \end{array}$$

where *W*_*p*_ is weight matrices. The $W_{p}\in {\mathbb{R}}^{m\times n}$ is masked by the pathway information matrix *M*∈ℝ^*m*×*n*^ with element-wise multiplication. Masking weights (*W*_*p*_°*M*) produce a sparse network by weighting between a particular pathway and genes. Genes not included in the pathway are set to zero. Note that non-zero weights of *W*_*p*_ are updated by backpropagation. *u*∈ℝ^*m*^ is a normalization vector to prevent the values of pathway nodes are affected by the number of genes in the pathway and is defined as follows:


(3) $$\begin{array}{l} u_{i}=\frac{1}{\sqrt{\overset{n}{\underset{j}{\sum }}M_{ij}}} \end{array}$$

For the fully connected nodes *f*, *n* input nodes are directly connected to the *f* through a linear layer as follows:


(4) $$\begin{array}{l} f=tanh\left(W_{f}g\right) \end{array}$$

where *W*_*f*_ is a weight matrix. *p* and *f* are concatenated after layer normalization ([*LayerNorm*(*p*); *LayerNorm*(*f*)]), and it passes through the hidden layer and the output layer. Then, output *o*∈ℝ is obtained. Tanh is used for the nonlinear transforming function, and batch normalization is utilized to decrease the effect of internal covariate shift induced by various weight scales. Dropout (α = 0.3) prevents overfitting of the model. We performed the training process by minimizing a cross entropy loss.

The training dataset was oversampled using SMOTE (Chawla et al., 2002) to avoid data imbalance. SMOTE oversampling creates synthetic instances and generates a new instance in line, a segment of the randomly selected *k*-nearest neighborhood of the minority class. To train PINNet, we initialized all weights with the Xavier initialization. We optimized the loss using the ADAM optimizer, the ReduceLROnPlateau scheduler, and a stochastic gradient descent algorithm, with a mini-batch size of 64. Note that the same settings are applied to both PINNet and the neural network model used for the comparison. The model was implemented using Python (version 3.7.4, https://www.python.org/) and Pytorch (version 1.8.1, https://www.pytorch.org/) on GeForce TITAN X GPUs.

### 2.4. Interpretation of PINNet and calculation of importance scores

We interpreted the model to identify highly contributing genes and pathways using the SHAP (SHapley Additive exPlanations) values. The SHAP values for each model were computed using the DeepExplainer SHAP package in Python, which is based on Deep SHAP (Lundberg and Lee, 2017). DeepExplainer calculates the feature importance of a given input to predict the deep neural network. The SHAP value is close to 0 if the feature does not affect the model's expectation. Deep SHAP explains the difference between output *y* and reference output ȳ by the summation of the difference between inputs *x* and reference inputs $\bar{x}$. Reference output ȳ are determined by executing forward passes in the network under reference inputs $\bar{x}$. Let △*x*_*i*_ be the difference between input *x*_*i*_ and reference input $\bar{x_{i}}$ and $C_{△x_{i}}^{\left(l\right)}$ be the attribution of node *i* in the *l*th layer. Then, the difference between output *y* and ȳ can be explained by the summation of attribution scores of *x*_*i*_ in the input layer *l*:


(5) $$\begin{array}{l} \underset{i}{\sum }C_{△x_{i}}^{\left(l\right)}=y-ȳ \end{array}$$

Reference inputs and outputs can be chosen for a given problem, and we set the reference to a training set with a balanced label distribution, where the reference output is the average of the outputs of the training set. The attribution score calculated from Deep SHAP can be adapted to an approximation of SHAP values. To obtain the contribution of the genes in the input layer, *x*_*i*_ in Equation (5) is gene expression values in a test set. Furthermore, to compute the contribution of pathway nodes in the pathway layer, the values of pathway nodes *p* in Equation (3) are used as *x*_*i*_ in Equation (5). For each pathway and brain and blood datasets, ten models are constructed by 10-fold cross-validation. Absolute mean SHAP values are normalized as *z*-scores, and the *z*-scores are averaged for the ten models. We defined it as an importance score. Importance scores are used to evaluate the contribution of the genes and pathways. The steps for evaluating and analyzing highly contributed genes are depicted in Figure 1.

## 3. Results

### 3.1. Performance in AD prediction

We applied PINNet to brain gene expression data (Narayanan et al., 2014) and blood gene expression data from ADNI (Petersen et al., 2010) and constructed four models using two pathway datasets: blood with GO [blood (GO)], blood with KEGG [blood (KEGG)], brain with GO [brain (GO)], and brain with KEGG [brain (KEGG)]. While constructing a sparse pathway network, we normalized the pathway nodes with the size of pathways. To identify an appropriate normalization method, we tried three types of a normalization vector *u*: (1) *u* = 1 (without normalization), (2) *u* = 1/*n*, and (3) $u=1/\sqrt{n}$, where *n* is the number of genes in each pathway, and compared the importance score of pathway nodes (Details about an importance score are described in Methods). Without normalization, pathways with a larger number of genes had higher importance scores in all four models. The absolute values of the Pearson correlation coefficient between the numbers of genes in pathways and importance scores were strongly correlated in the blood (GO) (|*R*| = 0.43) and blood (KEGG) (|*R*| = 0.30). However, when normalizing with $\sqrt{n}$, |*R*| < 0.1 was obtained in four models. This implies that the number of genes in the pathway has almost no effect on the importance scores of the pathways through normalization (Supplementary Figure S1). Based on this result, the value of the pathway node was normalized with $\sqrt{n}$. We trained PINNet to predict the disease status of samples (see Methods) and tested its performance by 10-fold-cross-validation. In the cross-validation, each fold preserved the ratio of sample classes, and the ratios of AD to CN were 0.46 and 1.97 in blood and brain, respectively. A part of the training set was used as a validation set so that the ratio of training, validation, and test set was 8:1:1. During the training of PINNet, a validation set was used for early stopping and selection of the number of fully connected nodes and learning rates with grid search (Supplementary Table S1). We selected the hyperparameters that yielded the best AUC on the validation set in each fold. Early stopping was applied if the validation AUC did not increase by more than ten epochs during the training. The number of fully connected nodes in the pathway layer is a hyperparameter with a set of candidate values of 32, 64, and 128, smaller than the number of pathways. The number of nodes in the second hidden layer was fixed as 64. Candidate values for the learning rate are 0.0001, 0.0005, and 0.001. All compared methods were trained and tested on the same data splits.

With the brain gene expressions from the prefrontal cortex area (Narayanan et al., 2014), the area under the curve (AUC) values between true positives and false-positive rates were 0.9744 and 0.9763 with GO and KEGG pathways, respectively (Table 1). The previous study using the same dataset showed the accuracy of around 86.30–91.22% (Cheng et al., 2021) using several machine learning models, reporting that brain gene expressions are valuable to classifying AD patients. This was also confirmed in our study. With the blood gene expressions from ADNI, we obtained AUC values of 0.6355 and 0.6420 with GO and KEGG pathways, respectively (Table 1). Predictions using the blood gene expressions showed relatively lower classification performance than brain expression data. This range of performance was also reported in the previous study (Lee and Lee, 2020).

**Table 1:** Comparison of performance (AUC) of different methods.

	**SVM**	**RF**	**DNN**	**PINNet**	
				**GO**	**KEGG**
Blood	0.6174 ± 0.1116	0.5731 ± 0.1099	0.6144 ± 0.0789	0.6355 ± 0.1095	0.6420 ± 0.0811
Brain	0.9850 ± 0.0232	0.9554 ± 0.0332	0.9744 ± 0.0401	0.9744 ± 0.0329	0.9763 ± 0.0288

Performance comparison for different machine learning and deep learning models. The DNN is a control model for each pathway dataset, consisting of a fully connected layer. SVM, support vector machine; RF, random forest; DNN, deep neural network; GO, gene ontology; AUC, area under the receiver operating characteristic curve.

We compared PINNet with deep neural network (DNN), support vector machines (SVM), and random forest (RF) methods. The DNN model architecture is identical to PINNet with the same number of the first hidden layer, except that it does not apply a pathway binary mask. The DNN model consists of two fully connected layers without a pathway binary mask, and the number of nodes and learning rate of the first hidden layer were selected using grid search in the validation set. Since the range of nodes in the first hidden layer of PINNet was 200 (168 + 32) to 4,154 (4,026 + 128), the candidate nodes in the first hidden layer include 128, 512, 1,024, and 4,096, and the learning rate used the same set of candidate values as PINNet. Similarly, for SVM and RF, the combination of hyperparameters and kernel type with the highest AUC for the validation set was chosen within each fold. Sets of hyperparameter values searched in the validation and subsequently selected best hyperparameter values are provided in Supplementary Table S2. As shown in Table 1, our PINNet outperformed the SVM and RF models, achieving better classification performance on blood datasets. In the brain dataset, the SVM model showed the highest performances, although all models showed high prediction performances. Despite the extra parameters in DNN, which is a fully connected model, the performance of the DNN was lower than PINNet, especially for blood. Additionally, Table 2 shows F1 scores of PINNet and comparing models.

**Table 2:** Comparison of performance (F1-score) of different methods.

	**SVM**	**RF**	**DNN**	**PINNet**	
				**GO**	**KEGG**
Blood	0.5609 ± 0.0559	0.5465 ± 0.0409	0.5563 ± 0.0635	0.5907 ± 0.0762	0.5542 ± 0.0547
Brain	0.9695 ± 0.0282	0.9411 ± 0.0250	0.9657 ± 0.0327	0.9607 ± 0.0330	0.9639 ± 0.0321

Performance comparison for different machine learning and deep learning models. The DNN is a control model for each pathway dataset, consisting of a fully connected layer. SVM, support vector machine; RF, random forest; DNN, deep neural network; GO, gene ontology; AUC, area under the receiver operating characteristic curve.

### 3.2. Known AD-related genes are important features of PINNet

A major objective of the analysis of gene expressions for the disease is to associate changes in the transcript with changes in phenotype. To reveal such associations, we interpreted PINNet models for AD prediction. We identified which genes played an important role in prediction in the models learned from 10-fold-cross validation in Table 1. To determine the contribution of each gene in AD prediction, the feature importance of nodes in the input layer was measured by the SHAP value using the Deep SHAP method, a backpropagation-based deep neural network interpretation algorithm (see the Methods section). For each pathway (GO and KEGG) and each gene expression dataset (brain and blood), we calculated SHAP values from the trained PINNet models for each gene. We calculated the importance score as the average of the normalized SHAP values of the ten models. As a result, we obtained the importance scores of genes for each model of blood (GO), brain (GO), blood (KEGG), and brain (KEGG).

**Figure 2:**
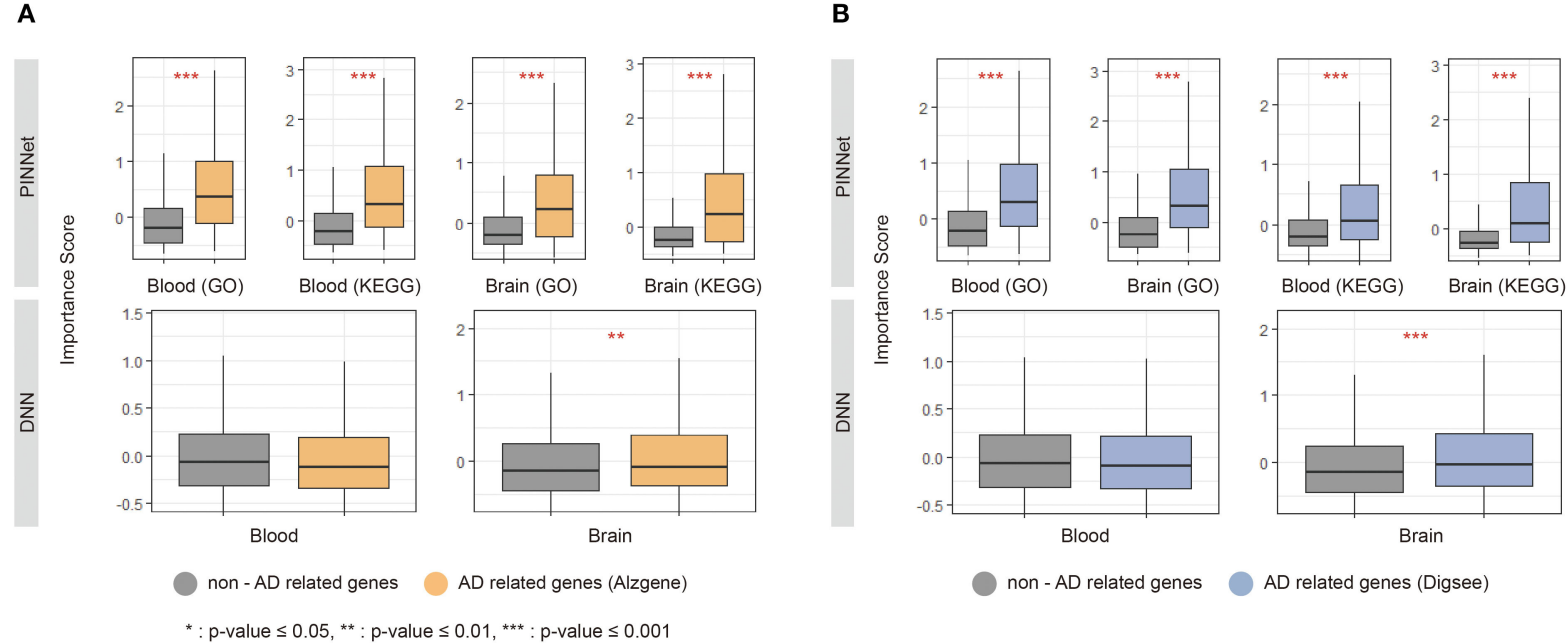
Importance score of known Alzheimer's disease (AD) genes in PINNet and DNN. We compared the SHAP (SHapley Additive exPlanations) values of known AD-related genes, including **(A)** Alzgene and **(B)** Digsee, in PINNet and DNN. The *y*-axes in each graph show the importance score. *Denotes a *p*-value ≤ 0.05 of the Wilcoxon test between non-AD related genes and AD-related genes; **indicates a *p*-value ≤ 0.01 of significance; ***represents a *p*-value ≤ 0.001.

We analyzed the importance scores of genes using two lists of known AD-related genes: AlzGene (Bertram et al., 2007) and DigSee (Kim et al., 2017). The AlzGene contains 681 genes curated by systematic meta-analyses, and 361 genes were shared with the 8,922 genes used in the study. DigSee contains 1,635 AD-related genes extracted from PubMed abstracts using text-mining techniques, and 961 were common with genes used in this study. We defined genes included in AlzGene or DigSee as AD-related genes and the remaining as non-AD-related genes. We used the Wilcoxon test to compare the importance score between AD-related and non-AD-related genes in PINNet and DNN. As shown in Figure 2, AD-related genes screened from DigSee and AlzGene showed significantly higher contributions than non-AD-related genes in PINNet models for all datasets (*p*-value ≤ 0.001). However, in the case of DNN models, the difference in importance score between AD-related genes and non-AD-related genes is only partially significant for brain data, and there is no difference in blood. In addition, we observed that the importance scores of known AD genes from PINNet were significantly higher than those from the DNN model for both KEGG and GO sources. These results shows the robustness of our approach. Unlike standard DNNs, PINNet captures important biological features based on prior biological knowledge. It represents that PINNet's biological prior knowledge leads to learning disease-related genes as essential features.

### 3.3. Finding sub-networks of highly contributed genes

**Figure 3:**
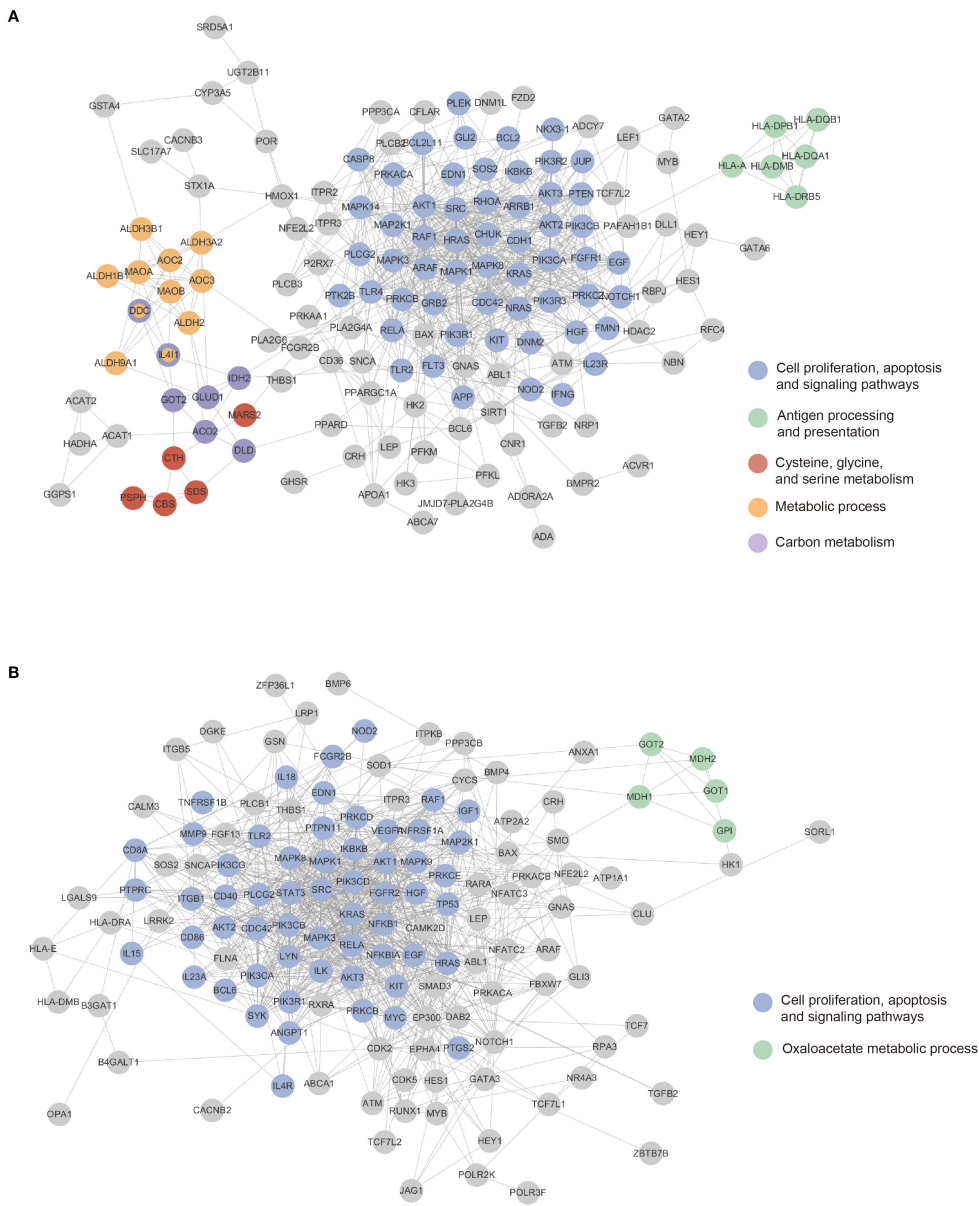
Mapping highly contributing genes to the PPI network. Colored nodes are sub-networks clustered using the ClusterOne algorithm. Gene nodes can be mapped to more than one sub-networks. **(A)** The blood sub-network. **(B)** The brain sub-network.

Using a protein interaction network obtained from the STRING database, we examined genes with high importance scores. We selected 1% genes with the highest importance scores from the four models. We integrated genes selected in PINNet (GO) and PINNet (KEGG) models for the blood and brain, separately, and obtained 170 and 167 genes in blood and the brain, respectively. The highly contributed genes were mapped onto the STRING database (version 11.0) (Szklarczyk et al., 2019), where interactions larger than a confidence score of 0.7 were used. Cytoscape (Shannon et al., 2003) was used to configure the PPI network. As shown in Figure 3, 152 and 138 genes in the blood and brain were mapped in the largest network, respectively (Supplementary Table S3). We also used ClusterOne (Nepusz et al., 2012) to screen PPI subnetwork modules. ClusterOne identifies overlapping functional modules by detecting dense regions in the protein interactome network. The parameters were set to *p*-values < 0.05, the minimum size = 5, and an edge weight as a confidence score in the STRING database. If genes of two subnetworks were overlapped by more than 70%, we merged them into a large module. Then, modules were selected if more than one biological term was enriched with GO BP and KEGG using DAVID (Sherman et al., 2009) (Bonferroni corrected *p*-values < 0.05). Figure 3 shows blue, green, red, orange, and purple modules in the blood and blue and green modules in the brain. In the blood, the blue module was significantly enriched in 218 terms related to proliferation and several signaling pathways, including Ras, FOXO, and PI3K-Akt signaling pathways. The green module was enriched with antigen processing and presentation terms, especially the MHC class II protein. In the red module, the serine family, comprising cysteine, glycine, and serine metabolism-related terms, was enriched. The orange module was related to several metabolic processes, including glycolysis, ethanol catabolic process, and aldehyde catabolic process. The purple module was enriched with several carbon metabolism pathways. In the brain, the blue module was similar to the blue module in blood. It was enriched with 350 terms related to proliferation and several signaling pathways, including TNF, FOXO, and PI3K-Akt signaling pathways. The green module contained 29 enriched terms related to the oxaloacetate metabolic process (Details about enrichment tests are in Supplementary Table S3).

### 3.4. Comparisons of contributions of genes on blood and brain dataset

We also identified highly contributed genes that show differences between blood and brain in AD prediction. First, we compared models based on the same pathway database (GO, KEGG) in different tissues with a two-sided Wilcoxon test (see Supplementary Table S4; Supplementary Figures S2, S3). From the results of 10-fold cross-validation, absolute SHAP values with *z*-score normalization were used for the test, and the *p*-values were corrected by Benjamini–Hochberg method (BH). We obtained 24 and 291 significantly different genes between blood and brain models (blood (GO) vs. brain (GO) and blood (KEGG) vs. brain (KEGG), respectively) with the adjusted *p*-values (BH) < 0.05. Next, we checked highly contributed genes (top 1%) that showed a clear distinction between the tissues. As a result, in the brain, CLU, PTPN11, ITPKB, PRKACB, IGF1, and MTHFD2 were more important compared to blood. CLU is related to several pathological states of AD, including a mediator of Aβ toxicity (Foster et al., 2019). PTPN11 (also known as SHP2) interacts with tau in Alzheimer's disease brain (Kim et al., 2019). Overexpression of ITPKB induces the increase of Aβ40 and tau hyperphosphorylation in a mouse model of familial Alzheimer's disease (Stygelbout et al., 2014). PRKACB is involved in the elevation of Aβ and tau hyperphosphorylation levels (Wang et al., 2019). Reduced IGF-1 signaling in the AD mouse model is attributed to a decline in neuronal loss and behavioral impairment (Cohen et al., 2009). MTHFD2 is differentially expressed in AD posterior cingulate astrocytes (Sekar et al., 2015). In the case of blood, although two genes were significantly important compared to brain, there is no known evidence to explain the relevance of these genes and AD in blood.

### 3.5. Prediction performance of genes with high importance scores

We further investigated whether genes selected using the importance scores in PINNet can be relevant features for predicting AD. First, we divided all samples into ten sets; eight sets were used for training, a set for selecting genes (named a feature selection set), and a remaining set for testing (see Supplementary Figure S4). Second, after training PINNet using the training set, we selected 892 (10%) genes with the highest importance scores in the feature selection set. Third, a multilayer perceptron (MLP) model was trained with the 892 selected feature genes, and then the remaining test set was used to evaluate the AD prediction performance. We repeated this process ten times. The MLP model consists of four layers, and the numbers of nodes in each layer are {*n*, *n*/2, *n*/4, 2}, where *n* is the number of input genes. We compared the performance of 10% of selected genes using PINNet with the same number of randomly selected genes and all genes for predicting AD samples. Genes selected using PINNet outperformed the random gene set in blood models (Table 3), suggesting that only 10% of the genes selected from PINNet can represent the whole blood transcriptome dataset.

**Table 3:** Performance comparisons (AUC) using all genes, random genes and selected genes from PINNet.

	**All (8,922)**	**Random (892)**	**Selected features (892)**	
			**PINNet (GO)**	**PINNet (KEGG)**
Blood	0.6092 ± 0.0762	0.5745 ± 0.0875	0.5938 ± 0.0776	0.6031 ± 0.0598
Brain	0.9764 ± 0.0258	0.9750 ± 0.0440	0.9720 ± 0.0343	0.9743 ± 0.0314

AUC, area under the curve; DNN, deep neural network.

## 4. Discussion

In this study, we developed PINNet, a model with higher interpretability than a black box neural network with pathway information as prior knowledge. When designing a model, relationships between pathways and genes were represented as weights between the input layer and the pathway layer.

We compared the performance of our proposed model with those of previous studies using blood and brain datasets. In the case of the brain dataset, prior studies employing the Weighted Gene Co-expression Network Analysis method for biomarker selection achieved an AUC ranging from 0.959 to 0.972 (Deng et al., 2021). Our model exhibits performance similar to that of the selected biomarker without an additional feature selection process. In the case of the blood dataset, Khanal et al. (2021) reported a 0.65 AUC in 5-fold cross-validation for the same dataset, which incorporated education and age features. PINNet's performance is comparable, even without the feature selection step and the inclusion of education and age features. Furthermore, PINNet provides more extensive insights into feature contributions compared to alternative methods. Our results show that, when contrasted with a deep neural network without pathway information, PINNet proves adept at learning informative groups of features, as depicted in Figure 2.

We further examined the importance score of pathway nodes in the pathway layer using the same ten models that analyzed the importance scores of input genes (Supplementary Table S5). The maximum values of importance scores for input gene nodes and pathway nodes were 11.904 and 1.512, respectively, and the variances were 0.697 and 0.321, respectively. Since the importance scores were defined as the average of *z*-scores of absolute mean SHAP values, this result means that there were relatively few pathway nodes having high importance scores across all ten models. Furthermore, there was no significant difference in importance scores of pathway nodes between blood and brain models. When the Wilcoxon test was performed, adjusted *p*-values (BH) were >0.05 for all pathways (Supplementary Table S5).

Although the average importance scores of pathway nodes were relatively smaller than compared to those of gene nodes, pathways with high importance scores were related to AD. The characteristics of the highly ranked pathways in the blood were related to the glycosylphosphatidylinositol (GPI) anchor-related pathway and immune response. The GPI anchor metabolic process was the highest-ranked GO BP, and similar KEGG pathways, such as glycerophospholipid metabolism and GPI-anchor biosynthesis, were also highly ranked among pathway nodes of blood (KEGG). In addition, several immune-related terms have been ranked top in blood. In recent studies, inflammation has been identified as an important contributor to AD pathology. Peripheral immune cells, such as T cells, are activated and infiltrate the inflamed brain area through the damaged blood-brain barrier (BBB) and accumulate in the AD brain (Togo et al., 2002; Town et al., 2005). Moreover, disrupted BBB may allow complement proteins to reach the brain from the plasma, and cell damage and death can lead to complement activation of neurons and oligodendrocytes, which activate complement and lead to dysregulation (Morgan, 2018).

In the prediction of the brain dataset, the GO BP term with the highest importance score is the regulation of filopodium assembly. Filopodia is the protrusion at the end of the neuron and is related to neural plasticity (Ozcan, 2017). AD pathology shows a correlation with filopodia density (Boros et al., 2019). In KEGG, neurodegenerative diseases such as Parkinson's disease, Alzheimer's disease, and prion disease showed high importance scores. SNARE interactions in vesicular transport ranked 9th on pathway nodes of the brain (KEGG), and it is associated with neurotransmitter release (Han et al., 2017). In particular, Aβ oligomers interfere with SNARE-medicated vesicle fusion, which may cause synaptic dysfunctions (Yang et al., 2015). Similar to blood models having pathways related to immune response, many immune-related pathways were also important in the brain models. We suggest that immune-related genes have the potential to advance the understanding of AD in terms of systemic disease in future studies. In future, external validation using additional clinical data could be pursued. As demonstrated in this study, the application of PINNet can provide valuable understanding of the underlying molecular mechanisms and biological processes.

## 5. Conclusion

We proposed a pathway information-based neural network model for the AD prediction. PINNet applied pathway-level of prior biological knowledge in constructing connections between genes and the AD status. We improved classification accuracies of AD and CN in blood and brain using PINNet, and the interpretation of the trained model revealed biological systems related to AD. Additionally, PINNet would be a promising model for various disease prediction tasks due to its possibility of model interpretability.

## Data availability statement

The original contributions presented in the study are included in the article/Supplementary material, further inquiries can be directed to the corresponding author.

## Author contributions

HL initiated and supervised the study. YK developed the method and performed the experiments. All authors participated in writing the manuscript and have read and approved the manuscript.

## References

[B1] Ashburner M. Ball C. A. Blake J. A. Botstein D. Butler H. Cherry J. M.. (2000). Gene ontology: tool for the unification of biology. Nat. Genet. 25, 25–29. 10.1038/75556 10802651 PMC3037419

[B2] Bateman R. J. Xiong C. Benzinger T. L. Fagan A. M. Goate A. Fox N. C.. (2012). Clinical and biomarker changes in dominantly inherited alzheimer's disease. N. Engl. J. Med. 367, 795–804. 10.1056/NEJMoa1202753 22784036 PMC3474597

[B3] Bertram L. McQueen M. B. Mullin K. Blacker D. Tanzi R. E. (2007). Systematic meta-analyses of alzheimer disease genetic association studies: the alzgene database. Nat. Genet. 39, 17–23. 10.1038/ng1934 17192785

[B4] Boros B. D. Greathouse K. M. Gearing M. Herskowitz J. H. (2019). Dendritic spine remodeling accompanies alzheimer's disease pathology and genetic susceptibility in cognitively normal aging. Neurobiol. Aging 73, 92–103. 10.1016/j.neurobiolaging.2018.09.003 30339964 PMC6251733

[B5] Chawla N. V. Bowyer K. W. Hall L. O. Kegelmeyer W. P. (2002). Smote: synthetic minority over-sampling technique. J. Artif. Intell. Res. 16, 321–357. 10.1613/jair.95324088532

[B6] Cheng J. Liu H.-P. Lin W.-Y. Tsai F.-J. (2021). Machine learning compensates fold-change method and highlights oxidative phosphorylation in the brain transcriptome of Alzheimer's disease. Sci. Rep. 11, 1–13. 10.1038/s41598-021-93085-z 34211065 PMC8249453

[B7] Cheng Y. Tian D.-Y. Wang Y.-J. (2020). Peripheral clearance of brain-derived aβ in alzheimer's disease: pathophysiology and therapeutic perspectives. Transl. Neurodegener. 9, 1–11. 10.1186/s40035-020-00195-1 32381118 PMC7204069

[B8] Cohen E. Paulsson J. F. Blinder P. Burstyn-Cohen T. Du D. Estepa G.. (2009). Reduced igf-1 signaling delays age-associated proteotoxicity in mice. Cell 139, 1157–1169. 10.1016/j.cell.2009.11.014 20005808 PMC3017511

[B9] Deng Y. Zhu H. Xiao L. Liu C. Liu Y.-L. Gao W. (2021). Identification of the function and mechanism of m6a reader igf2bp2 in Alzheimer's disease. Aging 13, 24086. 10.18632/aging.203652 34705667 PMC8610118

[B10] Durinck S. Spellman P. T. Birney E. Huber W. (2009). Mapping identifiers for the integration of genomic datasets with the r/bioconductor package biomart. Nat. Protoc. 4, 1184. 10.1038/nprot.2009.97 19617889 PMC3159387

[B11] Foster E. M. Dangla-Valls A. Lovestone S. Ribe E. M. Buckley N. J. (2019). Clusterin in Alzheimer's disease: mechanisms, genetics, and lessons from other pathologies. Front. Neurosci. 13, 164. 10.3389/fnins.2019.00164 30872998 PMC6403191

[B12] Han J. Pluhackova K. Böckmann R. A. (2017). The multifaceted role of snare proteins in membrane fusion. Front. Physiol. 8, 5. 10.3389/fphys.2017.00005 28163686 PMC5247469

[B13] Huang D. W. Sherman B. T. Lempicki R. A. (2009). Systematic and integrative analysis of large gene lists using david bioinformatics resources. Nat. Protoc. 4, 44–57. 10.1038/nprot.2008.211 19131956

[B14] Huynh R. A. Mohan C. (2017). Alzheimer's disease: biomarkers in the genome, blood, and cerebrospinal fluid. Front. Neurol. 8, 102. 10.3389/fneur.2017.00102 28373857 PMC5357660

[B15] Kanehisa M. Goto S. (2000). Kegg: kyoto encyclopedia of genes and genomes. Nucleic Acids Res. 28, 27–30. 10.1093/nar/28.1.27 10592173 PMC102409

[B16] Khanal S. Chen J. Jacobs N. Lin A.-L. (2021). “Alzheimer's disease classification using genetic data,” in 2021 IEEE International Conference on Bioinformatics and Biomedicine (BIBM) (Houston, TX: IEEE), 2245–2252.

[B17] Kim J. Kim J.-J. Lee H. (2017). An analysis of disease-gene relationship from medline abstracts by digsee. Sci. Rep. 7, 1–13. 10.1038/srep40154 28054646 PMC5215527

[B18] Kim Y. Liu G. Leugers C. J. Mueller J. D. Francis M. B. Hefti M. M.. (2019). Tau interacts with shp2 in neuronal systems and in alzheimer's disease brains. J. Cell Sci. 132, jcs229054. 10.1242/jcs.229054 31201283 PMC6679582

[B19] Kuenzi B. M. Park J. Fong S. H. Sanchez K. S. Lee J. Kreisberg J. F.. (2020). Predicting drug response and synergy using a deep learning model of human cancer cells. Cancer Cell 38, 672–684. 10.1016/j.ccell.2020.09.014 33096023 PMC7737474

[B20] Lai K. S. P. Liu C. S. Rau A. Lanctôt K. L. Köhler C. A. Pakosh M.. (2017). Peripheral inflammatory markers in Alzheimer's disease: a systematic review and meta-analysis of 175 studies. J. Neurol. Neurosurg. Psychiatry 88, 876–882. 10.1136/jnnp-2017-316201 28794151

[B21] Lee S. Lim S. Lee T. Sung I. Kim S. (2020). Cancer subtype classification and modeling by pathway attention and propagation. Bioinformatics 36, 3818–3824. 10.1093/bioinformatics/btaa203 32207514

[B22] Lee T. Lee H. (2020). Prediction of Alzheimer's disease using blood gene expression data. Sci. Rep. 10, 1–13. 10.1038/s41598-020-60595-1 32103140 PMC7044318

[B23] Li H. Hong G. Lin M. Shi Y. Wang L. Jiang F.. (2017). Identification of molecular alterations in leukocytes from gene expression profiles of peripheral whole blood of Alzheimer's disease. Sci. Rep. 7, 1–10. 10.1038/s41598-017-13700-w 29070791 PMC5656592

[B24] Lim B. Y. Lai K. W. Haiskin K. Kulathilake K. Ong Z. C. Hum Y. C.. (2022). Deep learning model for prediction of progressive mild cognitive impairment to Alzheimer's disease using structural mri. Front. Aging Neurosci. 14, 560. 10.3389/fnagi.2022.876202 35721012 PMC9201448

[B25] Lundberg S. M. Lee S.-I. (2017). A unified approach to interpreting model predictions. Adv. Neural Inf. Process. Syst. 30, 4765–4774.

[B26] Luo F. Wang M. Liu Y. Zhao X.-M. Li A. (2019). Deepphos: prediction of protein phosphorylation sites with deep learning. Bioinformatics 35, 2766–2773. 10.1093/bioinformatics/bty1051 30601936 PMC6691328

[B27] Mao W. Zaslavsky E. Hartmann B. M. Sealfon S. C. Chikina M. (2019). Pathway-level information extractor (plier) for gene expression data. Nat. Methods 16, 607–610. 10.1038/s41592-019-0456-1 31249421 PMC7262669

[B28] Morgan B. P. (2018). Complement in the pathogenesis of Alzheimer's disease. Semin. Immunopathol. 40, 113–124. 10.1007/s00281-017-0662-9 29134267 PMC5794825

[B29] Morris J. K. Honea R. A. Vidoni E. D. Swerdlow R. H. Burns J. M. (2014). Is alzheimer's disease a systemic disease? Biochim. Biophys. Acta 1842, 1340–1349. 10.1016/j.bbadis.2014.04.012 24747741 PMC4126236

[B30] Mostavi M. Chiu Y.-C. Huang Y. Chen Y. (2020). Convolutional neural network models for cancer type prediction based on gene expression. BMC Med. Genom. 13, 1–13. 10.1186/s12920-020-0677-2 32241303 PMC7119277

[B31] Narayanan M. Huynh J. L. Wang K. Yang X. Yoo S. McElwee J.. (2014). Common dysregulation network in the human prefrontal cortex underlies two neurodegenerative diseases. Mol. Syst. Biol. 10, 743. 10.15252/msb.20145304 25080494 PMC4299500

[B32] Nepusz T. Yu H. Paccanaro A. (2012). Detecting overlapping protein complexes in protein-protein interaction networks. Nat. Methods 9, 471–472. 10.1038/nmeth.1938 22426491 PMC3543700

[B33] Ozcan A. S. (2017). Filopodia: a rapid structural plasticity substrate for fast learning. Front. Synaptic Neurosci. 9, 12. 10.3389/fnsyn.2017.00012 28676753 PMC5476769

[B34] Petersen R. C. Aisen P. Beckett L. A. Donohue M. Gamst A. Harvey D. J.. (2010). Alzheimer's disease neuroimaging initiative (adni): clinical characterization. Neurology 74, 201–209. 10.1212/WNL.0b013e3181cb3e25 20042704 PMC2809036

[B35] Puthiyedth N. Riveros C. Berretta R. Moscato P. (2016). Identification of differentially expressed genes through integrated study of Alzheimer's disease affected brain regions. PLoS ONE 11, e0152342. 10.1371/journal.pone.0152342 27050411 PMC4822961

[B36] Sekar S. McDonald J. Cuyugan L. Aldrich J. Kurdoglu A. Adkins J.. (2015). Alzheimer's disease is associated with altered expression of genes involved in immune response and mitochondrial processes in astrocytes. Neurobiol. Aging 36, 583–591. 10.1016/j.neurobiolaging.2014.09.027 25448601 PMC4315763

[B37] Senior A. W. Evans R. Jumper J. Kirkpatrick J. Sifre L. Green T.. (2020). Improved protein structure prediction using potentials from deep learning. Nature 577, 706–710. 10.1038/s41586-019-1923-7 31942072

[B38] Shannon P. Markiel A. Ozier O. Baliga N. S. Wang J. T. Ramage D.. (2003). Cytoscape: a software environment for integrated models of biomolecular interaction networks. Genome Res. 13, 2498–2504. 10.1101/gr.1239303 14597658 PMC403769

[B39] Stygelbout V. Leroy K. Pouillon V. Ando K. D'Amico E. Jia Y.. (2014). Inositol trisphosphate 3-kinase b is increased in human alzheimer brain and exacerbates mouse alzheimer pathology. Brain 137, 537–552. 10.1093/brain/awt344 24401760

[B40] Subramanian A. Tamayo P. Mootha V. K. Mukherjee S. Ebert B. L. Gillette M. A.. (2005). Gene set enrichment analysis: a knowledge-based approach for interpreting genome-wide expression profiles. Proc. Nat. Acad. Sci. U. S. A. 102, 15545–15550. 10.1073/pnas.0506580102 16199517 PMC1239896

[B41] Szklarczyk D. Gable A. L. Lyon D. Junge A. Wyder S. Huerta-Cepas J.. (2019). String v11: protein-protein association networks with increased coverage, supporting functional discovery in genome-wide experimental datasets. Nucleic Acids Res. 47, D607–D613. 10.1093/nar/gky1131 30476243 PMC6323986

[B42] Togo T. Akiyama H. Iseki E. Kondo H. Ikeda K. Kato M.. (2002). Occurrence of t cells in the brain of alzheimer's disease and other neurological diseases. J. Neuroimmunol. 124, 83–92. 10.1016/S0165-5728(01)00496-9 11958825

[B43] Town T. Tan J. Flavell R. A. Mullan M. (2005). T-cells in Alzheimer's disease. Neuromol. Med. 7, 255–264. 10.1385/NMM:7:3:255 16247185

[B44] Urayama A. Moreno-Gonzalez I. Morales-Scheihing D. Kharat V. Pritzkow S. Soto C. (2022). Preventive and therapeutic reduction of amyloid deposition and behavioral impairments in a model of Alzheimer's disease by whole blood exchange. Mol. Psychiatry 27, 4285–4296 10.1038/s41380-022-01679-4 35835859 PMC10601825

[B45] Wang L. Liu J. Wang Q. Jiang H. Zeng L. Li Z.. (2019). Microrna-200a-3p mediates neuroprotection in alzheimer-related deficits and attenuates amyloid-beta overproduction and tau hyperphosphorylation via coregulating bace1 and prkacb. Front. Pharmacol. 10, 806. 10.3389/fphar.2019.00806 31379578 PMC6658613

[B46] Xing X. Yang F. Li H. Zhang J. Zhao Y. Gao M.. (2021). “An interpretable multi-level enhanced graph attention network for disease diagnosis with gene expression data,” in 2021 IEEE International Conference on Bioinformatics and Biomedicine (BIBM) (Houston, TX: IEEE), 556–561.

[B47] Xu M. Zhang D.-F. Luo R. Wu Y. Zhou H. Kong L.-L.. (2018). A systematic integrated analysis of brain expression profiles reveals yap1 and other prioritized hub genes as important upstream regulators in alzheimer's disease. Alzheimers Dement. 14, 215–229. 10.1016/j.jalz.2017.08.012 28923553

[B48] Yang Y. Kim J. Kim H. Y. Ryoo N. Lee S. Kim Y.. (2015). Amyloid-β oligomers may impair snare-mediated exocytosis by direct binding to syntaxin 1a. Cell Rep. 12, 1244–1251. 10.1016/j.celrep.2015.07.044 26279571 PMC4955600

[B49] Yiannopoulou K. G. Papageorgiou S. G. (2020). Current and future treatments in alzheimer disease: an update. J. Cent. Nerv. Syst. Dis. 12, 1179573520907397. 10.1177/1179573520907397 32165850 PMC7050025

[B50] Zhang R. Miller R. G. Madison C. Jin X. Honrada R. Harris W.. (2013). Systemic immune system alterations in early stages of Alzheimer's disease. J. Neuroimmunol. 256, 38–42. 10.1016/j.jneuroim.2013.01.002 23380586 PMC3641776

