# Supplementary material for: PINNet: a deep neural network with pathway prior knowledge for Alzheimer's disease
Source: Front Aging Neurosci. 2023 Jul 14;15:1126156. doi: 10.3389/fnagi.2023.1126156 (PMC10380929; doi:10.3389/fnagi.2023.1126156)

Supplementary Material

PINNet: Inferred Alzheimer's disease pathology by a deep neural network with pathway prior knowledge

Yeojin Kim[a], Hyunju Lee [a,b,] *

[a] School of Electrical Engineering and Computer Science, Gwangju Institute of Science and Technology, Gwangju, South Korea.

[b] Artificial Intelligence Graduate School, Gwangju Institute of Science and Technology, Gwangju, South Korea.

*Corresponding author

Hyunju Lee, PhD

Professor, School of Electrical Engineering and Computer Science

Professor, Artificial Intelligence Graduate School

Gwangju institute of science and technology

123 Chemdangwagi-ro, Gwangju, Korea

Postal zip-code: 61005

Tel: (+82) 62-725-2213; Fax: (+82) 62-715-2204

E-mail: hyunjulee@gist.ac.kr

Supplementary Figures

Supplementary Figure 1. Comparison of Importance Score between Pathway Nodes Normalization We compare Pearson correlation between number of genes of pathway and importance score. R is sample Pearson correlation coefficient. Normalization vector u is (1) $u_i = 1$ (without normalization), (2) $u_i = \frac{1}{\sum_j^n M_{ij}}$ (normalize by N), and (3) $u_i = \frac{1}{\sqrt{\sum_j^n M_{ij}}}$, where pathway information matrix is M (see Method).

KEGG, Kyoto Encyclopedia of Genes and Genomes database; GO, Gene ontology

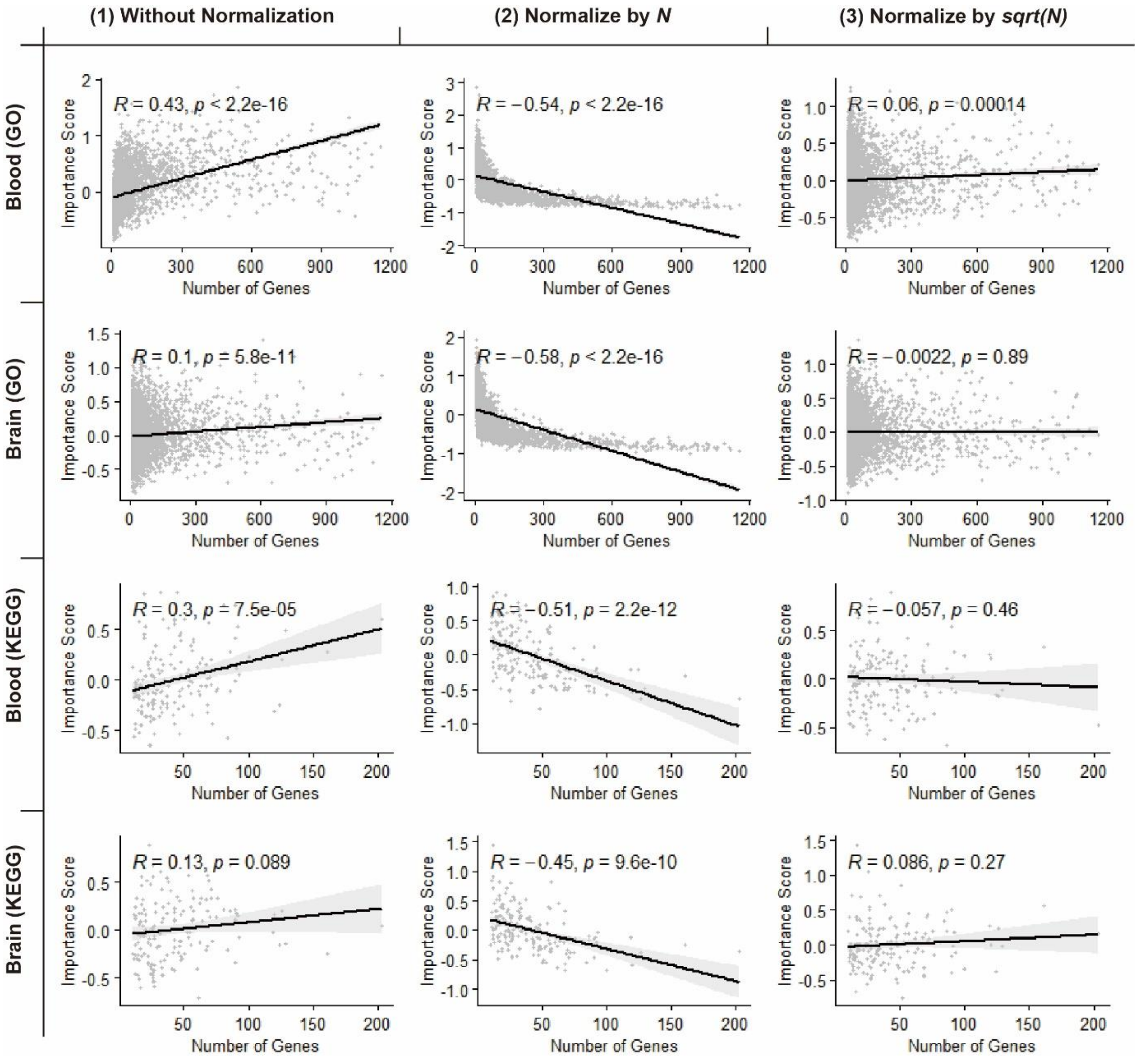

Supplementary Figure 2. Comparison of importance score of blood (GO) and brain (GO). x axis is importance score. We identified that 26 genes have significantly different contribution in blood (GO) brain (GO) through the Wilcox test (adjusted p-value (BH) < 0.05).

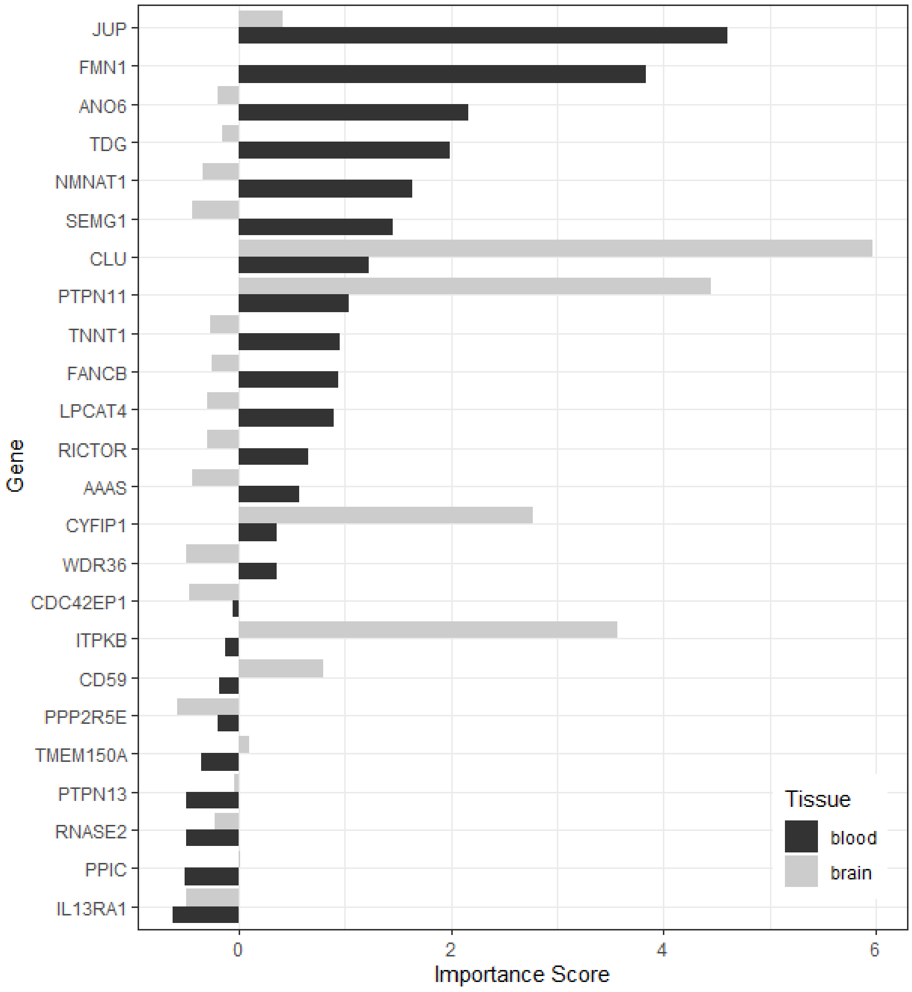

Supplementary Figure 3. Comparison of importance score of blood (KEGG) and brain (KEGG). x axis is importance score. We identified that 46 genes have significantly different contribution in blood (KEGG) brain (KEGG) through the Wilcox test (adjusted p-value (BH) < 0.01).

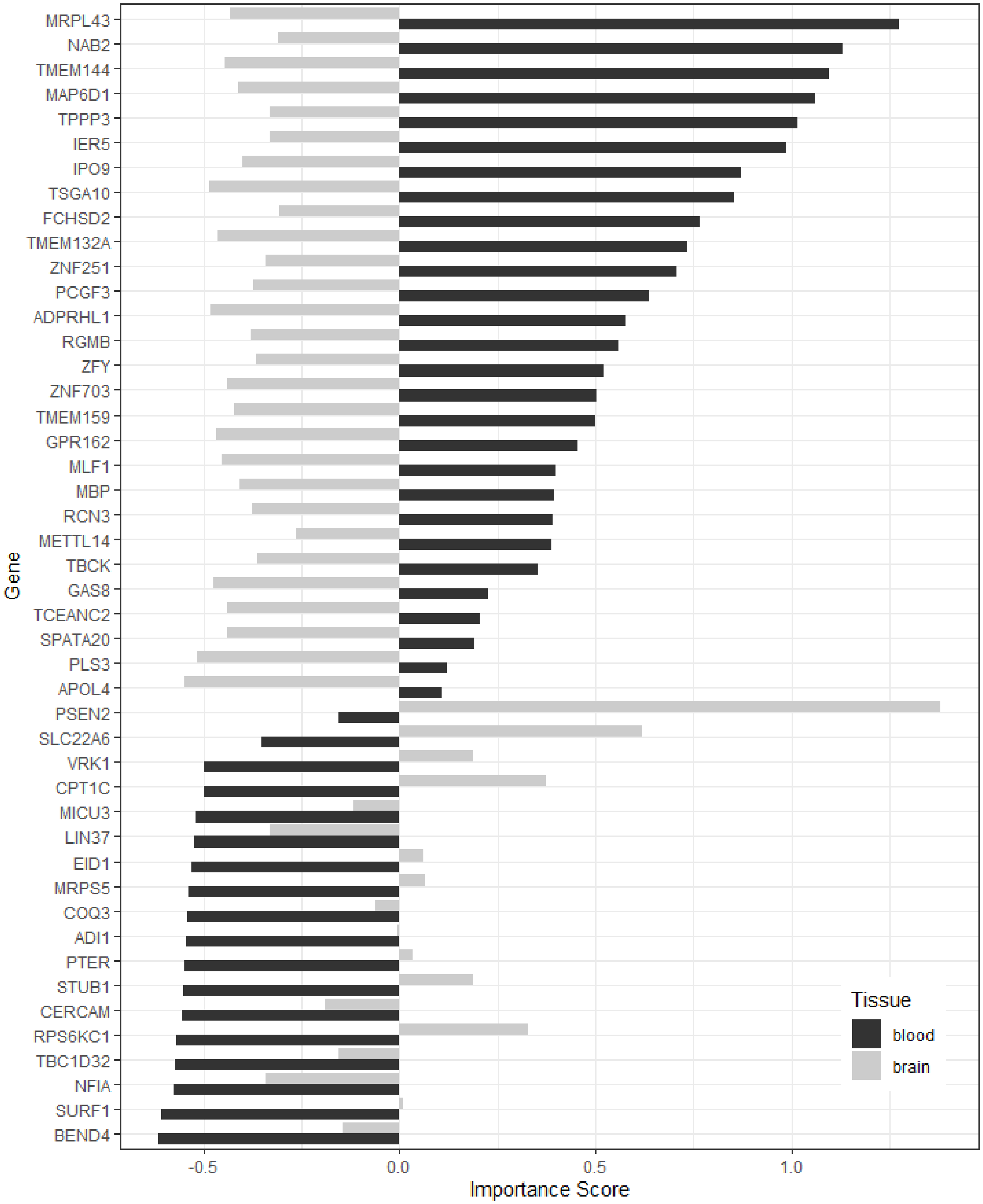

Supplementary Figure 4. Splitting data for checking the performance of highly contributed genes. We divide the entire data using 10-fold cross-validation by 8:1:1=training : validation for selecting genes : test. We trained a multilayer perceptron (MLP) model with 892 (10%) of highest importance genes and tested it on the test set.

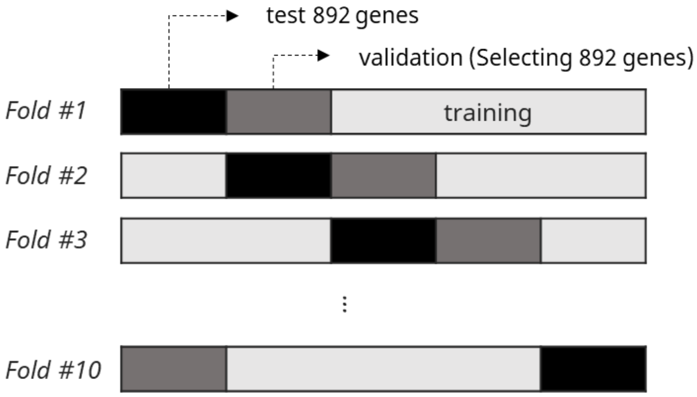

Supplement: Supplementary file 6 [file Data_Sheet_1.PDF]
